# Spontaneous Pneumothorax as an Atypical Presentation of Pulmonary Paracoccidioidomycosis: A Case Report with Emphasis on the Imaging Findings

**DOI:** 10.1155/2010/961984

**Published:** 2010-06-20

**Authors:** Mariana Leite Pereira, Edson Marchiori, Gláucia Zanetti, Guilherme Abdalla, Nina Ventura, Carolina Pesce Lamas Constantino, Viviane Brandão, Pedro Martins, Rodrigo Canellas, Antonio Muccillo, Romulo Varella de Oliveira

**Affiliations:** Department of Radiology, Rio de Janeiro Federal University, CEP 21941.913 Rio de Janeiro, Brazil

## Abstract

We describe the case of a 45-year-old male with pulmonary paracoccidioidomycosis and spontaneous pneumothorax. The patient presented to the hospital with sudden and intense chest pain accompanied by dyspnea and had a six-month history of dry cough, weight loss, and progressive dyspnea on exertion. Chest X-ray showed a small right pneumothorax, bilateral nonhomogeneous opacities, and emphysematous areas in the lung base. Chest computed tomography showed consolidation in both lungs, with architectural distortion, nodules, interlobular septal thickening, and emphysema, in addition to the right pneumothorax. A lung biopsy revealed yeast consistent with *Paracoccidioides brasiliensis*. No drainage was needed, and the lung was re-expanded. The patient was treated with antifungal drugs, showed mild improvement, and was referred to outpatient care.

## 1. Introduction

Paracoccidioidomycosis(PCM), caused by the fungus *Paracoccidioides brasiliensis,* is the most common systemic mycosis in South America [1, 2]. The disease is acquired by inhalation of infectious particles that develop into the primary infection in the lung. PCM has two clinical forms: a primary form with an acute or subacute clinical course and a chronic or reactivation form, with a more insidious course. The lung is the main target organ, and impact on its function is the main cause of morbidity and mortality in these patients [1–4]. 

 Chest radiography findings for PCM are linear and reticular opacities, nodules of various sizes, patchy ill-defined infiltrates, air-space consolidation, and cavitation [3]. However, chest X-ray offers limited evaluation for diffuse lung diseases. High-resolution computed tomography (HRCT) has become the method of choice for evaluating patients with pulmonary PCM. Nonetheless, although this disease is a common cause of pulmonary complications in Latin America, only a few reports contain HRCT findings for PCM. The most commonly described abnormalities are interlobular septal thickening, ground-glasses opacities, nodules, peribronchovascular interstitial thickening, and traction bronchiectasis [3–6]. 

 The aim of this work was to describe the thoracic imaging findings for a patient with confirmed pulmonary PCM who presented with a spontaneous pneumothorax. To our knowledge, this is the first description in the English medical literature of a pneumothorax secondary to paracoccidioidomycosis.

## 2. Case Presentation

**Figure 1:**
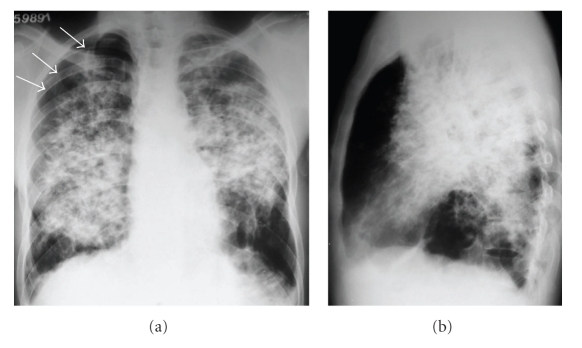
Chest radiographs in anteroposterior (a) and lateral (b) incidences showing a small right pneumothorax (arrows), and bilateral nonhomogeneous opacities, predominantly in the middle third of both lungs, with posterior predominance. Emphysematous areas are seen in the lung bases.

A 45-year-old man presented to the hospital with a four-hour history of sudden, intense chest pain on the right side, accompanied by marked dyspnea. He had a six-month history of progressive dyspnea on exertion, with a dry cough, and weight loss of 22 kg over this period. He also had a two-month history of unmeasured daily episodes of fever. The patient smoked 15 packs year. Physical examination revealed a thin, acyanotic patient with tachypnea. Other findings included a PA of 120 × 90 mmHg, HR of 112 bpm, and FR of 32 ipm. Reduced breath sounds were observed on the right side, with crackles in the upper two-thirds of both lungs. The rest of the physical examination was normal. Chest X-ray showed a small right pneumothorax, bilateral nonhomogeneous opacities that were predominantly in the middle third of both lungs, with posterior predominance, and emphysematous areas in the lung bases (Figure 1). Blood count, glucose, urea, creatinine, and liver function tests were normal. Serology for human immunodeficiency virus was negative. 

**Figure 2:**
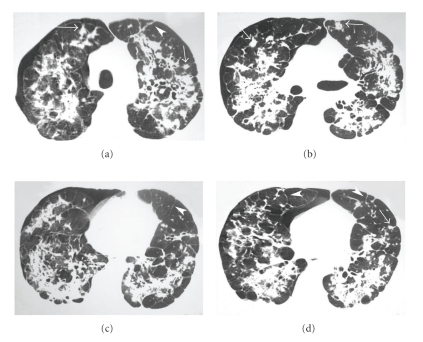
HRCT scans at four different levels showing areas of consolidation in both lungs, with signs of architectural distortion, irregular nodules (arrows), interlobular septal thickening (arrowheads), and emphysema, in addition to the right pneumothorax.

 A chest HRCT detected the right pneumothorax and consolidation in both lungs, with architectural distortion, irregular nodules, interlobular septal thickening, and emphysema (Figure 2). No chest tube drainage was required, and the lung was re-expanded. Bronchoscopy showed no structural changes. Direct examination and cultures for mycobacteria and fungi in the bronchoalveolar lavage were negative, and neoplastic cells were absent. Transbronchial biopsy showed no granulomas or malignant cells. Respiratory function evaluation showed mild mixed respiratory disorder, with a negative bronchodilator test. Open lung biopsy was performed and showed a chronic granulomatous inflammatory process. The examination of fresh samples revealed yeast consistent with *Paracoccidioides brasiliensis*. After treatment with sulfamethoxazole and trimethoprim, the patient showed a partial improvement for dyspnea and was referred to outpatient care.

## 3. Discussion

 PCM is the most frequent endemic mycosis in Latin America and is seen most commonly in Brazil, Argentina, Colombia, and Venezuela. However, several cases of PCM have also been reported in Europe and North America, mainly among immigrants and travelers from Latin America [7]. The areas with the most severe endemic PCM are the subtropical regions of Brazil, with infection particularly prevalent among farm workers. In these endemic areas, PCM is estimated to infect up to 10% of the population [2, 5, 6]. 

 The etiologic agent, *P. brasiliensis*, is an aerobic dimorphic fungus with an unknown habitat [4]. The disease is acquired by inhalation of infective particles that cause a self-limited, inflammatory parenchymal lung infection. The initial lesion is similar to the primary complex of tuberculosis, and is either controlled by natural defensive mechanisms or progresses to symptomatic disease. Following primary complex formation, the fungus can spread by the lymphatic system or blood circulation to the kidneys, spleen, liver, bone, adrenal glands, and central nervous system. Two main clinical forms of the disease are recognized: an acute form, and a localized or multifocal chronic form. The acute form occurs most commonly in young patients, and involves mainly the reticuloendothelial system, while the chronic form is most prevalent in adult men, and has a predominant pulmonary and mucocutaneous distribution [3–5]. The lung is the most commonly affected organ, in 50–100% of cases, and is the site of lesions associated with the acute and chronic forms of the infection [4]. 

 Findings on chest radiography have been described as linear and reticular opacities, nodules of various sizes, patchy ill-defined infiltrates, air-space consolidation, and cavitation. No general agreement exists for the predominant radiographic abnormalities, which reflects the subjectivity of radiographic interpretations in cases of diffuse lung disease [3]. HRCT is superior to chest radiography in assessing the pattern and distribution of parenchymal abnormalities, but despite the epidemiological importance of PCM in Latin America, only a few studies have assessed its manifestations by chest HRCT [5–7]. 

 Funari et al. [3] related the most common HRCT findings in 42 patients with pulmonary PCM, describing interlobular septal thickening, nodular opacities, traction bronchiectasis, peribronchovascular interstitial thickening, areas of cicatricial emphysema, and centrilobular nodular opacities. This study included patients who had been treated for PCM infection before the computed tomography (CT) investigation. The findings showed a predominant bilateral and symmetrical distribution, affecting all lung zones. Souza et al. [4] reported the findings for 77 patients with untreated pulmonary PCM. The most frequent HRCT findings were ground-glass attenuation areas, small centrilobular nodules, parenchymal bands, areas of cicatricial emphysema, interlobular septal thickening, architectural distortion, the reversed halo sign, and cavitated nodules. Most were predominant in the peripheral and posterior regions, involved all lung zones, and showed discrete predominance in the middle zones. 

 Pneumothorax is the presence of air in the pleural space, and is most often caused by accidental or iatrogenic trauma. Traditionally, in the absence of a known cause, it is referred to as spontaneous. In these cases, it is classified into primary form, which has no identifiable etiology or secondary form, which is associated with underlying lung disease [8–10]. Three mechanisms are responsible for pneumothorax: communication between alveolar spaces and pleura direct or indirect communication between the atmosphere and the pleural space; or the presence of gas-producing organisms in the pleural space [8]. 

 Secondary spontaneous pneumothorax can develop as result of obstruction, infection, infarction, neoplasm, or diffuse lung disease. The most frequent underlying disorders are chronic obstructive pulmonary disease with emphysema, cystic fibrosis, tuberculosis, lung cancer, or HIV-associated *Pneumocystis jiroveci *pneumonia, as well as more rare disorders such as lymphangioleiomyomatosis or Langerhans cell histiocytosis [8, 9, 11]. Because lung function in these patients is already compromised, pneumothorax often presents a potentially life-threatening disease, requiring immediate action [8, 9]. 

 Chronic infectious lung disease, such as tuberculosis, coccidioidomycosis, allergic bronchopulmonary aspergillosis, or histoplasmosis, may present with pneumothorax. Pneumothorax secondary to tuberculosis often heralds severe and extensive pulmonary involvement by the infectious agent, and the onset of bronchopleural fistula and empyema. It occurs in approximately 5% of patients with postprimary tuberculosis, usually in severe cavitary disease [12]. The pathogenesis involves pleural caseous infiltrates that undergo liquefaction, resulting in pleural necrosis and rupture [12]. Chronic coccidioidomycosis may manifest as persistent or progressive consolidation, cavitation, adenopathy, effusion, multiple nodules, fibrosis, or bronchiectasis. Coccidioidal cavities may be either thin- or thick-walled, and may rupture into the pleural space, resulting in a spontaneous pneumothorax or a persistent bronchopleural fistula [13, 14]. The classic radiographic manifestation of allergic bronchopulmonary aspergillosis is the presence of central saccular bronchiectasis. These airway abnormalities may lead to areas of postobstructive atelectasis or air trapping and subsequent pneumothorax [15, 16]. 

 The HRCT findings in our patient were areas of consolidation with signs of architectural distortion, irregular nodules, interlobular septal thickening, and emphysema, in addition to the right pneumothorax. The pneumothorax related to PCM was probably caused by rupture of an air-containing space (emphysema with or without bulla formation, or a cavitated lesion). To our knowledge, this is the first description in the English clinical literature of a pneumothorax secondary to paracoccidioidomycosis. 

 In conclusion, paracoccidioidomycosis should be included in the group of chronic infectious diseases that may present as a spontaneous pneumothorax.
